# Acute Effect of the “Zero Point” Method on Muscle Thickness and Muscle Damage in Trained Men

**DOI:** 10.3390/sports12010006

**Published:** 2023-12-22

**Authors:** Thiago B. Trindade, Ragami C. Alves, Nuno Manuel Frade de Sousa, Charles Lopes, Bruno Magalhães de Castro, Thiago S. Rosa, Jonato Prestes

**Affiliations:** 1 Department of Physical Education, Catholic University of Brasilia, QS 07, Lote 01, Taguatinga, Brasilia 71966-700, DF, Brazil; nunosfrade@gmail.com (N.M.F.d.S.); brunodemagalhaes@gmail.com (B.M.d.C.); thiagoacsdkp@yahoo.com.br (T.S.R.); jonatop@gmail.com (J.P.); 2 Department of Physical Education, Federal University of Paraná, Curitiba 80050-540, PR, Brazil; ragami1@hotmail.com; 3 Department of Physical Education, Methodist University of Piracicaba, Piracicaba 13420-835, SP, Brazil; charles_ricardo@hotmail.com; 4 Faculty Adventist of Hortolândia, Hortolândia 13184-010, SP, Brazil

**Keywords:** resistance training, time under tension, muscle swelling, delayed-onset muscle soreness

## Abstract

The “zero point” method allows for lower intensities for an exercise session without impairing the total training volume. This study aimed to compare the effects of the “zero point” versus the traditional method on muscle responses and muscle damage in trained men. Fifteen experienced men (age: 27.7 ± 6.4 years; body mass: 78.4 ± 11.4 kg; height: 174.8 ± 4.9 cm; experience: 5.86 ± 4.7 years; relative bench press strength: 1.38 ± 0.17 kg·kg^−1^) were subjected to two exercise protocols in a randomized order and separated by a week. The traditional and “zero point” methods were applied in the bench press, with loads of 70% and 50% of one repetition maximum (1RM), respectively, for 10 sets until concentric failure, with 3-min intervals between sets. The zero point method displayed a higher number of repetitions and time under tension than the traditional method, with no difference in the total training volume, echo intensity, algometry, lactate, and myoglobin. For the muscle thickness, no differences between the groups were presented, except for the deltoid muscle thickness, in which a higher post-training volume was observed compared to traditional training. The “zero point” method increases the demand on the deltoid muscles in the bench press exercise, but not on the pectoralis and triceps brachii.

## 1. Introduction

Resistance training (RT) methods or systems derive from the combination of acute variables, such as volume, intensity, muscle action, duration of repetitions, rest intervals, training frequency, type, and order of exercises [1,2,3]. RT methods are frequently used to maximize neuromuscular adaptations [4]. The use of advanced RT methods by bodybuilders, weight lifters, and RT practitioners is justified in situations in which the indiscriminate increase in intensity and number of traditional sets by itself is no longer sufficient to promote significant responses in strength and muscle hypertrophy [5,6,7]. Some advanced RT methods are presented as strategies to increase metabolic stress, and are commonly associated with an increase in the time under tension (TUT) of the skeletal muscle [8]; however, the literature is controversial regarding the efficiency of these techniques compared to traditional models [4]. It seems particularly important to first understand how these methods induce acute physiological responses compared to traditional RT.

De Almeida et al. [9] reported that the use of two versions of the method called “sarcoplasma stimulating training” (SST) in trained individuals, with variation in the predominant type of contraction or in the rest interval between sets, promoted a greater increase in the thickness of the elbow flexor and extensor muscles, at the expense of a lower total training volume, when compared with a traditional RT model. In the study by Marshal et al. [10], 14 trained men performed 20 repetitions in the free squat exercise under three conditions, all with 80% of 1 RM: 5 sets of 4 repetitions, with 3 min of rest between sets; 5 sets of 4 repetitions, with 20 s of rest between sets; or, repetitions to failure followed by 20-s intervals until 20 repetitions were achieved. The authors observed greater recruitment based on the increase in the amplitude of the electromyographic signals of the knee, hip, and erector spinae extensor muscles, when comparing the experimental protocol to the other protocols. De Camargo et al. [11] concluded that the use of the tri-set method in the barbell bench press, machine bench press, and cable fly exercises, although conducted for a lower total training volume, resulted in significantly greater increases in efficiency and workload, internal load, as well as for the thickness of the pectoralis major muscle when compared with a traditional protocol in trained individuals. Taken together, these results emphasize a difference in acute response as a result of applying advanced RT methods, and reveal the useful application of these strategies for several goals in RT programs designed for trained individuals.

In this context, a new method, called “zero point—ponto zero”, has been disseminated by the Brazilian bodybuilder Fernando Sardinha as a strategy that allows for the maintenance of a high intensity of effort in the RT sessions of highly trained practitioners, even with the use of lower loads (load intensity) in isolated or multi-joint exercises. It is proposed that, at the end of the eccentric phases of the exercise, a brief pause in the movement (≥1 s) is performed before starting the subsequent concentric phase. The maneuver would hypothetically minimize the contribution of elastic structures present in muscles and tendons that are expanded at the end of the eccentric phase of the exercise [12,13], and, therefore, increase the need for active tension production by the muscle itself.

In the transition from the eccentric to the concentric phase, the muscles can use a portion of the elastic energy to increase the force production in the subsequent action, at the expense of lower energy expenditure and greater mechanical efficiency [14]. However, if the transition time is not short enough, the potential energy can be dissipated in the form of heat, and not converted into kinetic energy [13]. The use of brief pauses at the end of the eccentric phase of the exercise, as proposed for the “zero point” method, would imply an expected reduction in performance, if the same intensity used in the conventional execution of the exercise was used. It is not known to what extent the minimization of the contribution of elastic structures to the concentric phase of a multi-joint exercise would imply an alteration in the acute demand on agonist and synergist muscles; considering that, in the face of severe fatigue, a redistribution of workloads between the muscles involved in the same exercise is expected to occur [15,16,17].

It is believed that acute changes in skeletal muscle dimensions, such as “muscle swelling”, which can be observed with the aid of ultrasound, result from physiological active hyperemia resulting from increased blood flow due to the increased demand for oxygen and nutrients, as well as for the removal of metabolites [18,19]. Hirono et al. [20] reported a moderate association between the acute “muscle pump” after the first RT session in untrained individuals, and the chronic increase in the same measure after a training program. Damas et al. [21] suggest that early changes in skeletal muscle dimensions, especially observed in untrained individuals, may derive from transient edema induced by muscle damage. The authors suggest evaluating the echo intensity as a complementary measure of muscle edema, as the increase in this measure may be related, at least in part, with muscle edema induced by muscle damage, directly interfering with “muscle pump” [21].

Therefore, the aim of this study was to compare the acute effects of the “zero point” method versus a traditional resistance training (RT) protocol on the echo intensity, muscle thickness, and biochemical markers of muscle damage in trained individuals. The initial hypothesis would be that the “zero point” method would result in greater time under tension and a greater magnitude of acute increase in muscle thickness for both agonist and synergistic muscles, with no differences expected for echo intensity and markers of muscle damage versus the traditional method.

## 2. Methods

### 2.1. Participants

Fifteen trained men with a mean age of 27.7 ± 6.4 years of age, whose characteristics are presented in Table 1, participated in the present study. The sample was recruited by convenience from advertisements on social networks: WhatsApp, Instagram, and Telegram.

The inclusion criteria adopted in the study followed the requirements suggested by Santos Júnior et al. [22] for the classification of highly advanced individuals in RT, which are the following: (a) uninterrupted practice of RT in the last 3 years; (b) currently training; (c) previous experience of at least 3 years in RT; (d) “excellent” bench press execution technique (assessed by two professionals); and (e) relative strength, in the bench press, greater than 120% of the body mass. All of the participants were informed about the study procedures and voluntarily provided signed informed consent. The protocol was approved by the institutional ethics committee of the Catholic University of Brasilia—Brazil (protocol number: 5.177.624). The participants had no positive answers on the physical activity readiness questionnaire (PAR-Q) nor any history of musculoskeletal injury in the upper limbs. This study was performed in accordance with the Declaration of Helsinki.

The exclusion criteria were the following: (a) physical disability or musculoskeletal limitations that prevented the regular practice of RT; (b) vegetarians; (c) daily protein intake below 1.4 g per kg of body mass; (d) use of medication capable of affecting muscle hypertrophy or the ability to train intensely; (e) history of use of anabolic steroids in the 6 months preceding the beginning of the experimental period; (f) systematic practice of any other exercise/sport modality during this study; (g) carriers of any chronic degenerative disease. One participant was excluded based on the criterion of the technique of performing the bench press exercise, and another was excluded for not reaching 120% relative strength in the 1RM test in the same exercise.

### 2.2. Procedures

All of the data were collected in four sessions. In the first session, participants were assessed in terms of height, body mass, and completed forms; in the second, they performed the 1RM test, and 72 h later they repeated the procedure to test the reliability of the data obtained. In the third and fourth sessions, the experimental procedures were carried out. In these two sessions, all of the participants were subjected to two resistance exercise (RE) protocols, the “zero point” and the traditional, in randomized order, with a one-week rest interval between the protocols.

Before the exercise sessions, measurements of thickness, echo intensity, and pressure algometry were collected in the pectoralis major, sternal and clavicular portions; deltoid, clavicular portion; triceps brachii, lateral head; in addition to blood lactate concentration and myoglobin. During the RT sessions, the number of repetitions, time under tension, and total volume in each set were quantified. Five minutes after the last set in each session, measurements of blood lactate concentration, muscle thickness, and echo intensity were evaluated. On the days following the exercise sessions (24, 48, and 72 h later), the participants were again evaluated for muscle thickness and echo intensity, as well as algometry and myoglobin. The participants were instructed to maintain their usual diets, and were regularly asked about any dietary changes that might influence the study’s results, such as the use of dietary supplements or variations in protein or carbohydrate intake, and avoiding training. They were questioned about their diet at the end of each training session, and guidance was reinforced to maintain their usual diets. None of the participants reported changes at any of the times they were surveyed. All training sessions took place on Mondays between 1:00 pm and 3:00 pm.

#### 2.2.1. Resistance Exercise Protocols

The exercise sessions, regardless of the protocol, started with a specific warm-up set on the bench press (8 repetitions with 50% of 1RM). The load used to perform the “zero point” method was set at 50% of 1RM, as the pilot study indicated that most participants were unable to complete the protocol (10 sets) with higher loads (70, 60, or 55% of 1RM). For the traditional protocol, 70% of 1RM was used.

For both RE protocols, a cadence of 1 s was used for the concentric phase, and 2 s for the eccentric phase of the exercise, with no pause between the ascending and descending movements. For the execution of the “zero point”, a pause of 1 s was performed at the end of the eccentric phase in all repetitions (end of the descending phase of the bench press), before starting the concentric phase. The fixed cadences for each training protocol were controlled with the aid of a metronome. The repetitions were registered in every set, as long as the execution technique was preserved for the complete range of motion. A team composed of three qualified professionals supervised each participant individually during the entire execution of the training sessions.

The total training volume (TTV) of each session was calculated based on the following equation: TTV = number of sets × number of repetitions × weight [23]. The time under tension was measured in all sets with the aid of a Garmin digital stopwatch, model Fenix 3 HR (Taipei, Taiwan). The timer was started from the moment the bar was delivered by an assistant into the hands of the participant, and interrupted when muscle failure was obtained. Isometric muscle actions were considered for the calculation of time under tension.

#### 2.2.2. One Repetition Maximum Test

For the assessment of maximum dynamic strength, a standard 1RM test protocol was used, as previously documented by Baechle et al. [24]. Two tests were performed on different days with an interval of 72 h between them, following the same protocol: after a general warm-up (5 min on a cycle ergometer at light intensity, performed at the beginning of the session), the evaluated participants performed eight repetitions with 50% estimated load for 1RM; after two minutes of interval, three more repetitions were performed with 70% of 1RM. After three minutes of this last set, subsequent tests were performed for a maximum repetition, with progressively heavier loads, until the 1RM measurement was determined in a maximum of three attempts, using 3 to 5 min of rest between each attempt. The descriptions of Brown and Weir [25] were observed in the standardization of the range of motion and exercise technique. The intraclass correlation (ICC) between the tests was 0.98. The highest load obtained in the two tests was considered a measure for 1RM.

#### 2.2.3. Muscle Thickness and Echo Intensity

The muscle thickness (MT) analysis was performed using B-mode ultrasound (Medison SA-99000^®^, Live 4D; Sansung Medison Co., LTD; Gyeonggi-Do, Republic of Korea), with a 100 mm transducer, 10–15 MHz. The transducer was coated with water-soluble transmission gel, which facilitated acoustic contact with minimal depression of the skin surface. For the evaluation of the pectoralis major (sternocostal and clavicular portion) and deltoid (clavicular portion) muscles, ultrasound images at rest were recorded at a specific joint angle—30° of shoulder abduction—while the participants remained lying on a stretcher in a supine position for 20 min. To obtain the measurement of the triceps brachii (lateral head), the participants migrated to a prone position and kept the arm supported, elbow slightly flexed with the muscle relaxed. For all muscle groups, the transducer was positioned perpendicular to the tissue interface, without depressing the skin, and aligned with the superficial and deep aponeuroses. When the image quality was considered satisfactory, it was saved on the hard disk.

The dimensions of the MT were verified by measuring the distance from the adipose tissue–subcutaneous muscle interface to the muscle–bone interface, according to the methodology described by Abe et al. [26]. Measurements were taken on the right side of the body and standardized according to the following parameters:

Pectoralis major (PM—sternocostal portion): from 1/3 of the distance between the sternoclavicular joint and the axillary crease, between the third and fourth ribs;

Pectoralis major (PM—clavicular portion): 1/3 of the distance between the sternoclavicular joint and the axillary fold, between the clavicle and the aponeurosis of the clavicular bundles of the PM;

Deltoid (clavicular portion): 1/2 of the distance between the acromion and the deltoid tuberosity;

Triceps brachii (lateral head): 1/3 of the distance between the acromion and the lateral epicondyle of the humerus.

The MT measurements were quantified using ImageJ 1.42q image analysis software (National Institutes of Mental Health, Bethesda, MD, USA). All of the images were digitally analyzed. To maintain consistency between testing protocols (“zero point” and traditional), each site was marked with henna ink (reinforced during the week). In order to ensure greater measurement accuracy, at least 3 images were obtained of each of the anatomical points described. If the measurements were within 1 mm of each other, the average of the values was calculated to obtain a final measurement. The data are expressed in millimeters (mm). No significant differences were observed between measurements taken before the experimental protocols. The intraclass correction coefficient (ICC) for the thicknesses of the deltoid, PM clavicular portion, PM sternal portion, and lateral head triceps muscles were r = 0.99, r = 0.99, r = 1.00, and r = 0.99, respectively.

Image J 1.42q software was used to determine the mean echo intensity of a grayscale histogram (0, black; 256, white), calculated for the region of interest (ROI, 1 × 1 = 1 cm^2^), observing the procedures described by Chen et al. [27]. The relative change in echo intensity was calculated based on the value obtained pre-exercise.

#### 2.2.4. Myoglobin and Blood Lactate Concentration

For the determination of myoglobin in blood plasma, approximately 5 mL of venous blood was withdrawn via a venipuncture technique from the cubital fossa region of the participants. The plasma myoglobin concentration was measured with an automated clinical chemistry analyzer (Model Elecsys 2010, F. Hoffmann-La Roche Ltd., Tokyo, Japan), using a commercial test kit (Roche Diagnostics, Indianapolis, IN, USA). The blood lactate was analyzed using a Yellow Springs Instruments (Yellow Springs, OH, USA) model 2700 Select lactate analyzer, using the electro-enzymatic method. Biochemical measurements were performed in duplicates to minimize any abnormal variation.

#### 2.2.5. Muscle Pain

The muscle pain threshold was quantified with a pressure algometer (brand MED.DOR Ltd., Valadares, MG, Brazil) with maximum compression capacity = 50 kgf, accuracy = 0.1 kgf, and a 3-digit display. The equipment has a round rubber application surface, with an area of 1 cm^2^. A trained evaluator performed the pressure pain threshold collection in the same muscles and anatomical references that were evaluated with ultrasound.

The pressure pain threshold was collected before each experimental session and also on the subsequent 3 days to assess the behavior of delayed-onset muscle soreness. In all of the assessments, the following positioning was adopted: (1°) the participant remained seated with their feet on the floor; (2°) hands resting on their thighs; and (3°) with their torso upright. Each anatomical point received a progressive pressure of 1 kg/s controlled via a metronome, until the participant interrupted due to “unbearable” pain. At this moment, the examiner pressed the “tare” button to lock the reading, immediately retracting the pressure algometer. Then, the pressure pain threshold reading was recorded. Three measurements were taken for each location, 10–15 s apart, and the highest pressure value observed in the three attempts was considered. The same evaluator was responsible for all of the algometry measurements.

### 2.3. Statistical Analysis

The ANOVA two-way repeated measures statistical test was applied to determine the effect of different RE sessions over time on algometry, repetitions, TTV, time under tension, MT, lactate, myoglobin, and echo intensity [28]. Considering the non-normality for myoglobin, a logarithmic transformation was applied. The Shapiro–Wilk test was used to analyze the normality of the studentized residuals of each variable, and no residue showed deviations greater than ±3 standard deviations (SD). The sphericity analysis was performed using Machly’s test and, in case of violation, the Greenhouse–Geisser correction was used. In case of interaction, simple main effects analysis was applied and the Bonferroni correction was used. For the variable repetitions, the adjusted value of *p* was ≤0.005 (0.05/10 comparisons). For the time under tension, the adjusted value of *p* was ≤0.005 (0.05/10 comparisons).

For the ANOVA two-way repeated measures statistical test, the intragroup effect size was calculated for the muscle thickness variable. The omega squared (Ω2) recommended for small samples was used, and the values ≤ 0.01, 0.01–0.06, 0.06–0.14, and >0.14 were considered: trivial, small, medium, and large, respectively [29].

To calculate the technical measurement error (TEM) for the muscle thickness variable, the SD of test and retest differences was divided by √2 [30]. The TEMs for the deltoid muscle in the clavicular portion were 0.14 mm, 0.29 mm (PM clavicular portion), 0.47 mm (PM sternal portion), and 0.23 mm (triceps lateral head). Changes in muscle thickness with values ≤ 0.14 mm (deltoid clavicular portion), ≤0.29 mm (PM clavicular portion), ≤0.47 mm (PM sternal portion), and ≤0.23 mm (triceps lateral head) represented a TEM. The a posteriori sample power was calculated for the deltoid muscle thickness variable in the clavicular portion. Considering a difference between groups ≥0.33 mm [30,31,32] (clinical difference based on the multiplication of 0.2 × SD pre), the observed power was 0.52, effect size was 0.30, and the alpha was 0.05 for a total sample size of 28 participants. For the data analysis, SPSS software (version 20.00) and G*Power 3.1.6 [33] were used. An alpha level of ≤0.05 was used as the significant difference.

## 3. Results

Table 1 presents the anthropometric and strength characteristics of the participants.

### 3.1. Repetitions

**Figure 1:**
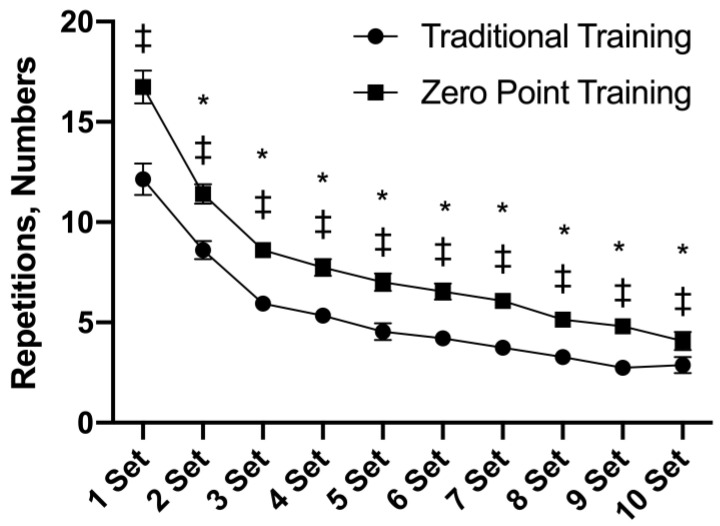
Repetitions presented as mean ± standard error. * *p* ≤ 0.05 vs. set 1. ‡ *p* ≤ 0.05 differences between groups for the same set.

Figure 1 shows the interaction for the repetitions F (9, 126) = 6.48, *p* = 0.001. A main effect of time was observed F (9, 126) = 167.21, *p* = 0.001. From set 1 to set 10, differences between the groups were observed, with a higher number of repetitions found for the “zero point” RE session (*p* = 0.001). The numbers of repetitions from set 2 to set 10 were significantly lower when compared to set 1 (*p* = 0.001).

### 3.2. Time under Tension (TUT)

**Figure 2:**
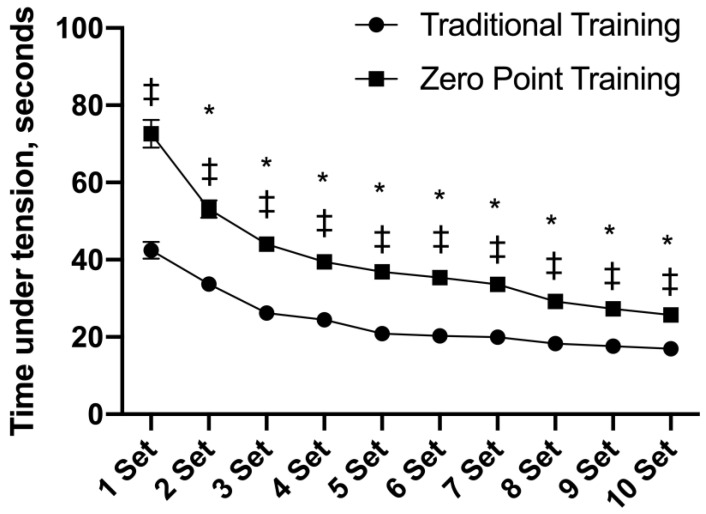
Values of time under tension presented as mean ± standard error. * *p* ≤ 0.05 vs. set 1. ‡ *p* ≤ 0.05 differences between groups for the same set.

Figure 2 shows the interaction for the TUT F (9, 126) = 17.54, *p* = 0.001. A main effect of time was observed F (9, 126) = 112.41, *p* = 0.001. From set 1 to set 10, differences between the groups were observed, with a higher TUT for the “zero point” RE session (*p* = 0.001). From set 2 to set 10, the TUT was lower when compared to set 1 (*p* = 0.001).

### 3.3. Total Training Volume (TTV)

**Figure 3:**
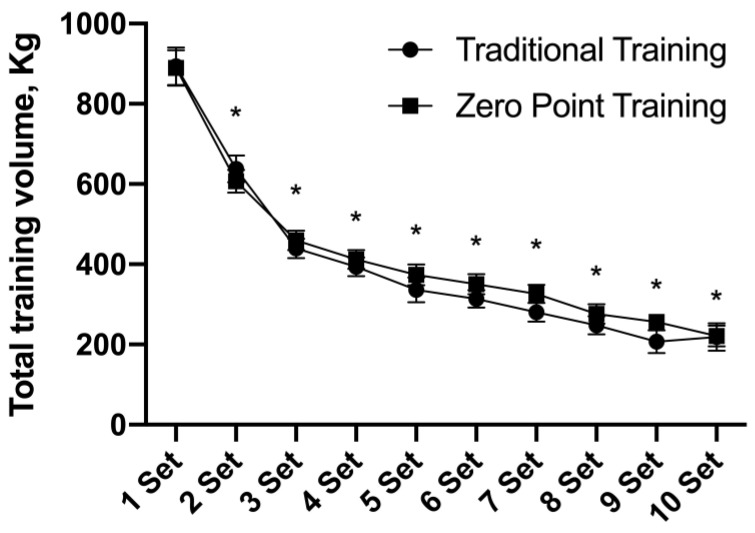
Total training volume presented as mean ± standard error. * *p* ≤ 0.05 vs. pre.

There was no interaction for the TTV F (9, 126) = 1.26, *p* = 0.26. However, a main effect of time was observed F (9, 126) = 202.50, *p* = 0.001. From set 2 to set 10, the TTV was lower when compared to set 1 (*p* = 0.001). See Figure 3.

### 3.4. Muscle Thickness (MT)

**Figure 4:**
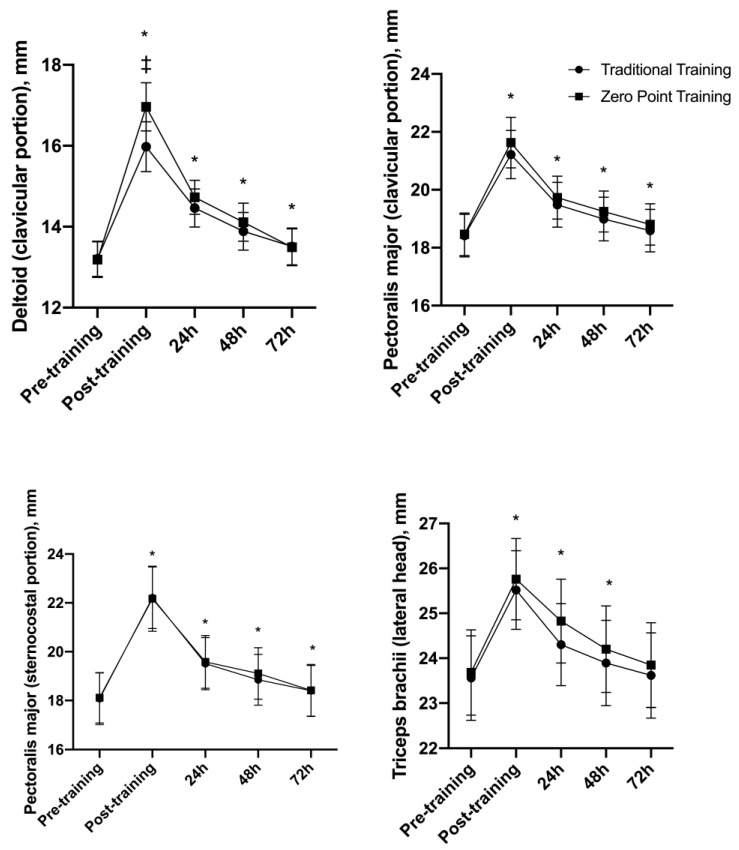
Muscle thickness presented as mean ± standard error. * *p* ≤ 0.05 vs. ‡ *p* ≤ 0.05 differences between groups at the same time point.

Figure 4 shows the interaction for the deltoid muscle F (4, 52) = 11.25, *p* = 0.001, with a main effect of time F (4, 52) = 151.93, *p* = 0.001. The moments after (*p* = 0.001), at 24 h (*p* = 0.001), at 48 h (*p* = 0.001), and at 72 h (*p* = 0.015) were significantly higher when compared to the moments pre-exercise. After the analysis of the simple main effects (simple main effects), the “zero point” RE session displayed higher values in the moment after when compared with the traditional RE session (*p* = 0.001). For the traditional and the “zero point” RE sessions, the Ω2 values were 0.22 (large) and 0.36 (large), respectively. The differences in the means at the moments after (+3.27 mm), at 24 h (+1.40 mm), at 48 h (+0.80 mm), and at 72 h (+0.30 mm) were above the TME (≤0.14). 

There was no interaction for the PM clavicular and external portion F (4, 56) = 0.57, *p* = 0.68 and F (4, 56) = 0.58, *p* = 0.67, respectively, while a main effect of time was observed for F (4, 56) = 85.11, *p* = 0.001 and F (4, 56) = 93.03, *p* = 0.001, respectively. The moments after (*p* = 0.001 for both), at 24 h (*p* = 0.002 and *p* = 0.001, respectively), at 48 h (*p* = 0.008 and *p* = 0.001, respectively), and at 72 h (*p* = 0.045 and *p* = 0.026, respectively) were higher when compared to the moments pre-exercise. For the traditional and the “zero point” RE sessions, the Ω2 values were 0.10 (mean) and 0.12 (mean), respectively. Differences in the means after (+2.98 mm), at 24 h (+1.16 mm), and at 48 h (+0.68 mm) were above the TME (≤0.29 mm) for the PM clavicular portion (Figure 4). For the PM external portion, the Ω2 values were 0.11 (mean) and 0.10 (mean), respectively. Differences in the means after (+4.09 mm), at 24 h (+1.45 mm), and at 48 h (+0.88 mm) were above the TME (≤0.47 mm).

There was no interaction for the triceps brachii lateral head F (4, 56) = 1.71, *p* = 0.16, while a main effect of time was observed F (4, 56) = 117.70, *p* = 0.001. The moments after (*p* = 0.001), at 24 h (*p* = 0.001), and at 48 h (*p* = 0.001) were higher when compared to the moments pre-exercise (Figure 4). For the traditional and “zero point” RE sessions, the Ω2 values were 0.04 (small) and 0.04 (small), respectively. Differences in the means after (+2.01 mm), at 24 h (+0.94 mm), and at 48 h (+0.42 mm) were above the TME (≤0.23 mm).

### 3.5. Echo Intensity (EI)

**Figure 5:**
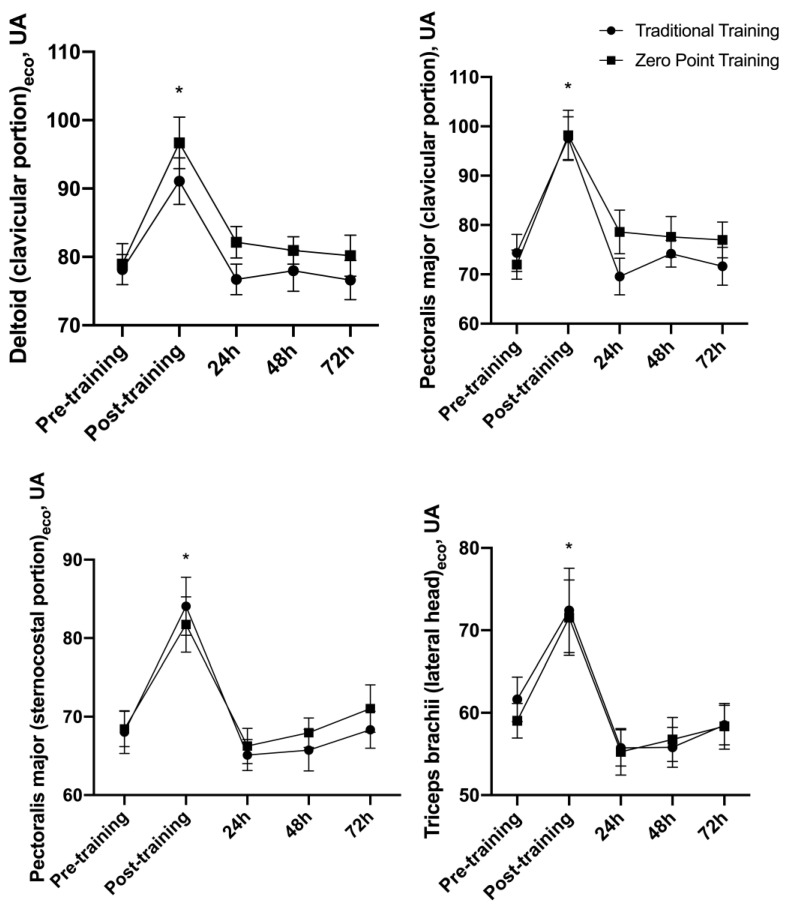
Echo intensity presented as mean ± standard error. AU = arbitrary units, * *p* ≤ 0.05 vs. pre.

There was no interaction for the EI of the clavicular deltoid muscle, PM clavicular and external portions, and triceps brachii lateral head F (4, 48) = 0.94, *p* = 0.44, F (4, 48) = 1.90, *p* = 0.12, F (4, 48) = 0.42, *p* = 0.79, and F (4, 48) = 0.53, *p* = 0.71, respectively. A main effect of time was observed F (4, 48) = 20.90, *p* = 0.001, F (4, 48) = 38.15, *p* = 0.001, F (4, 48) = 13.66, *p* = 0.001, and F (4, 48) = 16.61, *p* = 0.001, respectively. The moment after (*p* = 0.001) was significantly superior when compared to the moment pre-exercise for all muscles (Figure 5). For the traditional and “zero” point RE sessions, the Ω2 values were 0.21 (large) and 0.23 (large) for the clavicular deltoid muscle, respectively. The Ω2 values were 0.40 (large) and 0.21 (large) for the PM clavicular portion, respectively, and were 0.29 (large) and 0.18 (large), for the PM external portion, respectively. The Ω2 values were 0.21 (large) and 0.42 (large) for the triceps, respectively. See Figure 5.

### 3.6. Algometry

**Figure 6:**
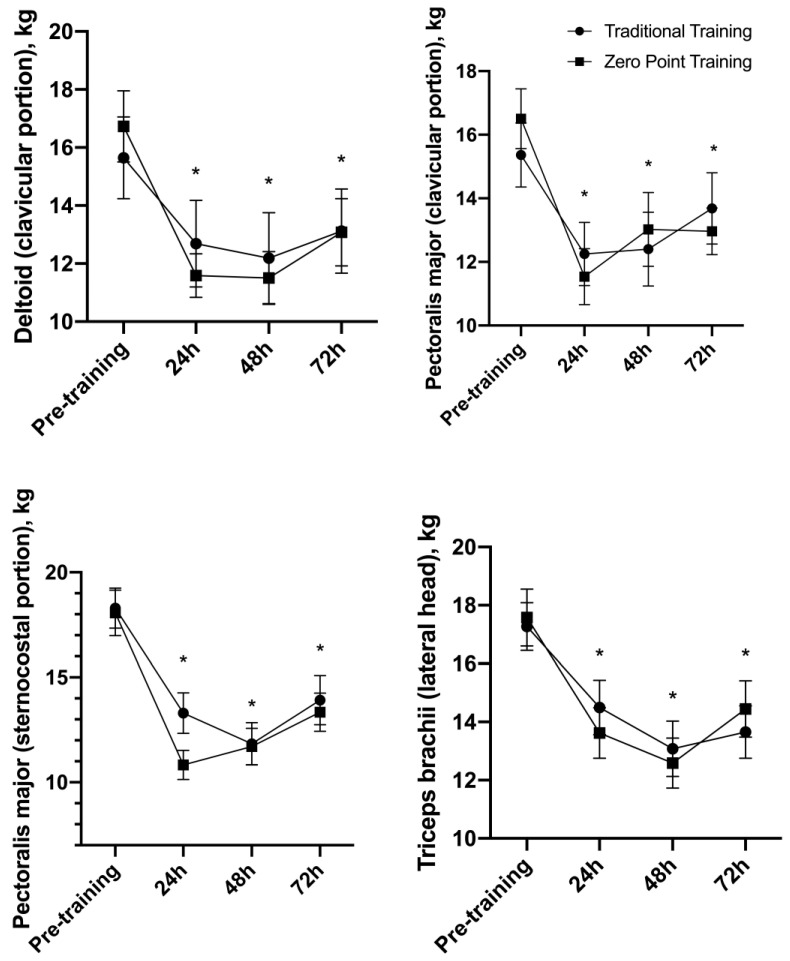
Algometric values before and after traditional and “zero-point” resistance exercise sessions (mean ± standard error). * *p* ≤ 0.05 vs. pre.

There was no interaction for the deltoid muscle, PM clavicular and external portions, and triceps brachii lateral head algometry F (3, 42) = 0.70, *p* = 0.55, F (3, 42) = 1.08, *p* = 0.36, F (3, 42) = 2.01, *p* = 0.12, and F (3, 42) = 0.89, *p* = 0.45, respectively. However, a main effect of time was observed F (3, 42) = 17.04, *p* = 0.001, F (3, 42) = 14.29, *p* = 0.001, F (3, 42) = 55.73, *p* = 0.001, and F (3, 42) = 20.11, *p* = 0.001, respectively (Figure 6). The 24 h (*p* = 0.001), 48 h (*p* = 0.002), and 72 h (*p* = 0.003) moments were significantly lower when compared to the pre-exercise moment. See Figure 6.

### 3.7. Lactate

**Figure 7:**
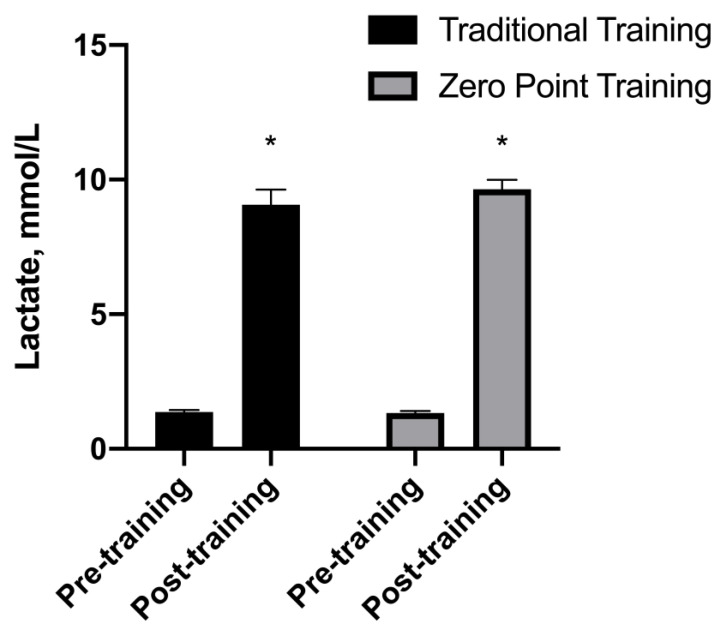
Lactate values presented as mean ± standard error. * *p* ≤ 0.05 vs. pre.

There was no interaction for the lactate F (1, 56) = 0.80, *p* = 0.37, while a main effect of time was observed F (1, 56) = 562.83, *p* = 0.001. The lactate levels were higher in the moment after compared to the moment pre-exercise. See Figure 7.

### 3.8. Myoglobin

**Figure 8:**
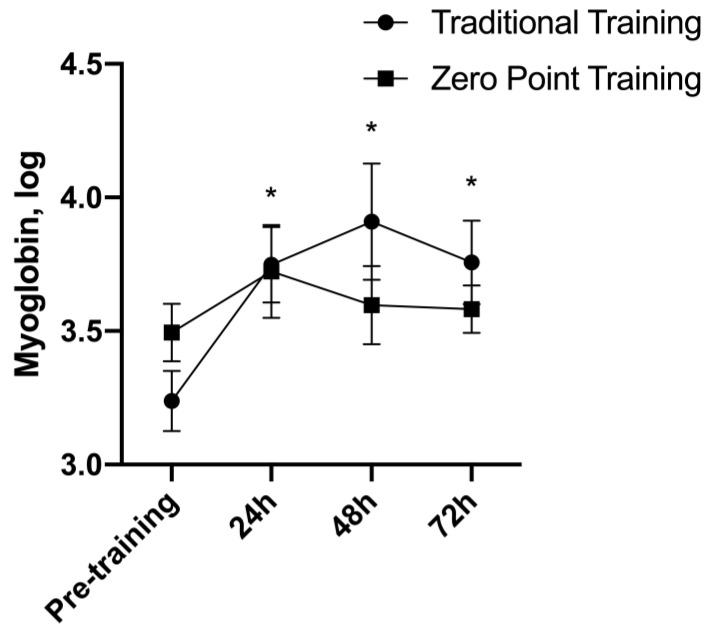
Myoglobin values presented as mean ± standard error. * *p* ≤ 0.05 vs. pre.

There was no interaction for the myoglobin F (3, 42) = 2.26, *p* = 0.09, while a main effect of time was observed F (3, 42) = 5.10, *p* = 0.004. The myoglobin levels were higher at the 24 h (*p* = 0.034), 48 h (*p* = 0.029), and 72 h (*p* = 0.004) moments compared to the moment pre-exercise (Figure 8). For the traditional and the “zero point” RE sessions, the Ω2 values were 0.12 (mean) and 0.00 (trivial), respectively. 

## 4. Discussion

The hypothesis that the “zero point” method would result in a greater TUT in an exercise session was confirmed in trained men, influenced by the greater number of repetitions obtained in all sets performed using the “zero point” method; meanwhile, no difference was found for the TTV and algometry. Although there was no interaction between the groups for myoglobin, the traditional protocol resulted in a greater magnitude of increase in relation to the “zero point”, considering the effect size. Contradicting the initial hypothesis, there was no difference between the training conditions for the MT of the agonist muscles, while the “zero point” session generated a greater increase in the deltoid muscle MT immediately after the session. In all of the evaluated regions, an increase in the echo intensity was observed, with no difference between the groups. Considering the effect size, the traditional protocol resulted in a greater magnitude of increase for the sternal and clavicular portions of the PM, while the “zero point” method promoted a greater magnitude increase for the lateral head of the triceps brachii. 

This study is the first to investigate the advanced RE method called “zero point”. The results are consistent with those of previous research that used similar procedures and instruments to evaluate other advanced RE methods. In the study by De Camargo et al. [11], the use of the tri-set method in the bench press exercises with a free barbell, machine bench press, and “fly” on the cable promoted greater swelling, based on the MT of the pectoralis major muscle in trained individuals when compared to a traditional RE session. Both protocols were conducted with the same intensity (10RM) for an equivalent number of sets, while the tri-set method resulted in a smaller volume load. The use of two variations of the SST method by De Almeida et al. [9] also resulted in a lower TTV in trained men. Higher acute increases in the thickness of elbow flexor and extensor muscles were achieved with SST versus the traditional protocol, with a similar increase in blood lactate concentrations assessed after RE sessions.

This evidence, combined with our results, strengthens the contemporary idea that advanced RT methods can serve as additional stimuli to break through plateaus and avoid the monotony of RT [5]. Although muscle hypertrophy is an outcome commonly prioritized by practitioners who choose to use these strategies, other approaches, such as reducing the volume or training intensity, or shortening the recovery between sessions, as long as they do not decrease the morphological responses, would justify the use of advanced methods. Such benefits could be obtained through strategies that incite greater physiological stress, factors considered important even to induce musculoskeletal hypertrophy [8].

The “zero point” method is based on the premise of delaying the transition time between the eccentric–concentric phases of the exercise, with the objective of minimizing the contribution of the elastic structures present in muscles and tendons, and thus increasing the demand on the agonist muscles. However, our results showed an increase in MT only for one synergist (deltoid) with the use of the “zero point” method compared to traditional RE. Contrary to our hypothesis, it seems that the reduction in the contribution of elastic structures may reflect a greater demand for synergistic muscles during multi-joint exercise, and does not potentiate the agonist. In addition to the pectoralis major muscle, the deltoid, especially in its clavicular portion, contributes significantly to the horizontal adduction of the shoulder, a common joint action when performing the bench press exercise. Therefore, a greater demand on the deltoid itself would be expected to guarantee the continuity of the task in the face of possible fatigue affecting the pectoralis major. Interestingly, the use of the “zero point” approach did not result in greater demand on the triceps brachii muscle, based on the thickness measurement, possibly due to the lesser involvement of the elbow extensor muscles in the bench press exercise, at least when compared with the muscles responsible for the horizontal adduction of the shoulder [34,35,36], although the lateral head is the most demanded portion of the triceps brachii in that exercise [34].

A possible explanation for this phenomenon may be a change in the management of the load sharing between the muscles involved in the exercise by the central nervous system, in this case, due to the lower use of elastic potential energy for the production of movement [34]. Previous studies conducted with isometric exercises support our results, through verifying changes in the EMG activity pattern during fatiguing contractions and clearly demonstrating the greater involvement of synergistic muscles during task performance [15,16]. This workload redistribution may be modulated by afferent feedback during certain conditions, such as fatigue, to maintain the task [17]—in our case, the repeated contractions.

Despite the suggested moderate correlation between the acute increase in the MT and chronic adaptations (ρ 0.443, þ 0.039) [20], Damas et al. [21] suggested that, in untrained individuals, the early increases induced by an RE session, for example, derive in large part from muscle swelling induced by edema, which can be evaluated by the echo intensity. Significant increases in the echo intensity, in addition to other muscle damage markers, can be observed in untrained individuals on the days following an exercise session [27,35,36]. In the present study, the increase in the measurement performed right after the session, but not in the days that followed it, reflects a lower susceptibility of trained individuals to the muscular damage resulting from an RE session [37].

However, the difference observed in the magnitude of the effect size for the echo intensity of the PM muscle, both in the sternal and clavicular portions, may be attributed to the tendency of traditional RE towards greater muscle damage in those specific regions. The opposite was observed in the lateral head of the triceps brachii, reinforcing the hypothesis of workload transfer to synergist muscles. The myoglobin behavior observed in the two conditions, an indirect marker of muscle damage [38], can also be attributed to the protocol of the traditional method and its greater predisposition to muscle damage, while no difference was observed in relation to the “zero point” method for the algometry measurements. This tendency goes against the initial hypothesis of the present study, since it was expected that the inclusion of an isometric action in long muscle length at the end of the eccentric phase of the bench press exercise would maximize the increase in the MT and echo intensity, given that muscle damage induced by isometric actions is more influenced by the muscle length than by the tension produced [36,39,40]. On the other hand, the faster recovery of the “zero point” method when compared to traditional RE can be useful for certain phases of the training program of experienced individuals.

Although this is the first study to investigate the acute effects of the “zero point” method, this study is not without limitations. Considering the possibility of regional structural variations, thickness measurements obtained in a muscle region do not necessarily represent the observable changes in the entire muscle. Furthermore, any extrapolations in relation to the findings of the present study must be conducted with caution, especially with regard to the chronic effects of the “zero point” method, as well as in other populations (women, elderly, high-level athletes), other methods, or different muscle groups. Finally, the limited number of participants should also be considered as a limitation.

## 5. Conclusions

Individuals who are experienced with RT can use brief pauses (1s) at the end of the eccentric phase of an exercise to increase the time under tension of the sets and allow for a reduction in the used load (from 70% to 50% 1 RM) without reducing the volume of training. This strategy enables better recovery in the days following the training session; although it results in similar levels of swelling in agonist muscles, it tends to increase the demand on synergist muscles during the bench press exercise. However, it is worth mentioning that modest differences were observed between both protocols for muscle swelling and markers of muscle damage.

## Figures and Tables

**Table 1:** Anthropometric and strength characteristics of the participants.

	Mean ± Standard Deviation
Age (years)	27.7 ± 6.4
Body mass (kg)	78.4 ± 11.4
Height (cm)	174.8 ± 4.9
BMI (kg/m^2^)	25.6 ± 2.8
1 RM (kg)	107.6 ± 16.8
1 RM relative (kg/kg)	1.38 ± 0.17
Training experience (years)	5.86 ± 4.7
Sets/week—bench press	8.4 ± 3.6
Sets/week—pectoralis major	25.8 ± 3.6

BMI, body mass index; RM, repetition maximum.

## Data Availability

Data is contained within the article. The data presented in this study are available in the main text.
